# Necrosis of the Tongue as a Late Complication of Radiotherapy

**DOI:** 10.22038/IJORL.2023.67124.3305

**Published:** 2023-09

**Authors:** Cristina Aguiar, Paulo Pina, Nuno Medeiros, Mónica Teixeira, Leandro Ribeiro, Pedro Oliveira

**Affiliations:** 1 *Department of Otorhinolaryngology, Centro Hospitalar Vila Nova de Gaia/Espinho, Porto, Portugal.*

**Keywords:** Late complications, Radiotherapy, Small-vessel disease, Tongue necrosis

## Abstract

**Introduction:**

Irradiation to treat head and neck cancer, causing chronic tissue damage, is associated with the development of vascular disease. Interest has risen over the effects of radiotherapy on major vessels, due to its high morbidity and mortality rate. However, small-vessel disease has been poorly studied and described.

**Case Report:**

We present a case of a patient with bilateral necrosis of the anterior third of the tongue, occurring 3 years after chemoradiotherapy treatment for squamous cell carcinoma of the floor of the mouth. Contrast-enhanced CT scan showed multiple areas of stenosis concerning both external carotid arteries and their branches, and total opacification of lingual arteries. Conservative management was performed, with auto-amputation on the fifth day, which allowed healing by secondary intention.

**Conclusions:**

Necrosis of the tongue appears as a rare late complication of radiotherapy, possibly due to its acceleration effect on the atherosclerosis process. Following small-vessel disease, one can assume a higher potential risk of major-vessel disease, highlighting the importance of a routine assessment and prophylaxis of thrombotic events.

## Introduction

Despite its fundamental role in the treatment of head and neck cancer, radiotherapy is associated with a large spectrum of bothersome to life-threatening events. 

As life expectancy continues to increase in cancer patients, there is a growing concern with treatment and, most importantly, prevention of late complications, such as vascular disease, mainly major vessel disease. The high cerebrovascular event rate observed in head and neck cancer patients may represent long-term treatment toxicity, imposed on patients with an already increased risk reflected by factors like smoking or male sex (1). Literature is scarce when it comes to effects of radiotherapy on small-vessels.

We present a case of bilateral tongue necrosis in a patient with a history of squamous cell carcinoma of the floor of the mouth treated with chemoradiotherapy.

## Case Report

A 51-year-old man underwent combined induction chemotherapy (3 cycles of docetaxel-cisplatin-fluorouracil) followed by consolidation chemotherapy (7 cycles of cetuximab) and radiotherapy (70Gy to the tumor bed and 50Gy to the neck) in 2018 for stage cT4a cN2c cM0 squamous cell carcinoma of the floor of the mouth, with complete remission of the disease clinically and radiologically on further otolaryngology follow-up appointments. The patient had a mild untreated dyslipidemia (total cholesterol > 218mg/dL) and active smoking. There was no history of hypertension, stroke or diabetes. In 2021, he presented himself at the emergency department with an 8-day history of glossodynia and pain-related dysphagia, with exclusive nutrition through his feeding tube. The patient denied symptoms of fever, arthralgia, neurologic alterations, weight loss, visual disturbances, jaw claudication, or temporal tenderness. There was no history of vasoconstrictor use, syphilis, cardiac arrest, or embolization.

**Fig 1:**
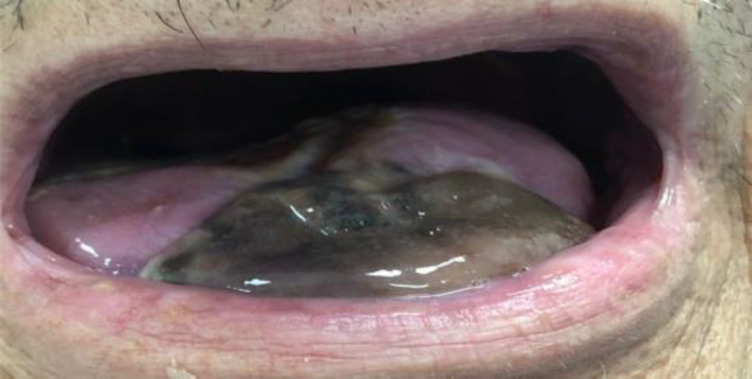
Necrosis of the anterior third of the tongue bilaterally on initial evaluation

Physical examination of the head and neck showed worsening of previously restricted tongue movements, highly painful at superficial palpation, and bilateral necrosis of the anterior third of the tongue (Figure 1). 

**Fig 2:**
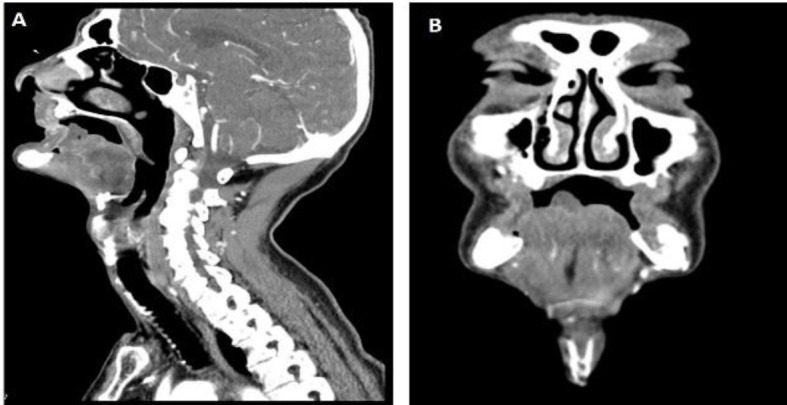
Contrast enhanced CT of the head and neck, sagittal (A) and coronal (B) views

Laboratory workup was normal, except for an elevation of both the C-reactive protein (2,51mg/dL) and erythrocyte sedimentation rate (60mm/hr). Serologies were negative for syphilis and other infections. Contrast-enhanced CT scan showed multiple areas of stenosis concerning both external carotid arteries and their branches, and total opacification of lingual arteries (Figure 2). A biopsy of the transition of necrotic to healthy tissue was performed, revealing unspecific inflammatory findings. 

**Fig 3:**
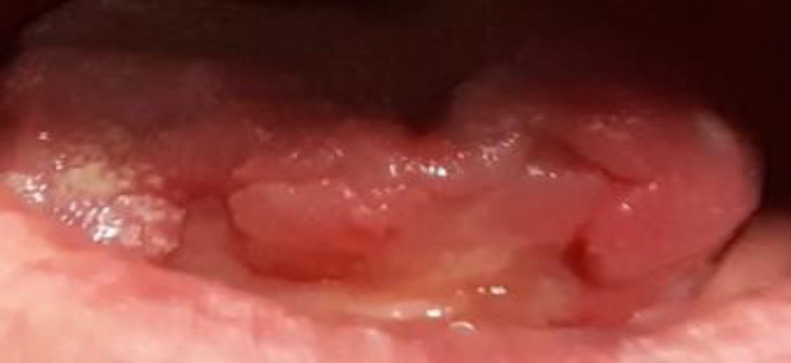
Complete recovery on the third month follow-up appointment

His condition was managed conservatively, with broad-spectrum antibiotics, while awaiting natural auto-amputation of the necrotic tissue, which occurred on the fifth day. The area healed by secondary intention. On the 3-month follow-up consultation, the patient denied glossodynia, which allowed him to further progress his oral uptake. The physical examination revealed total healing of the tongue (Figure 3).

## Discussion

We describe a case of bilateral tongue necrosis in a patient with a history of squamous cell carcinoma of the floor of the mouth treated with chemoradiotherapy (CR).

Standard treatment of locally advanced oral carcinomas (LAOC) includes surgical resection and radiotherapy or CR depending on the presence of nodal extracapsular spread and positive margins (2). Less frequently, primary CR has shown successful outcomes, in terms of locoregional control and survival rates, with a high cost of acute and late side effects (2).

Chemotherapy most common effects include kidney failure, hearing loss, tinnitus, nausea, vomiting and peripheral neuropathy (3), while radiotherapy’s most common effects include dysphagia, salivary gland dysfunction, osteoradionecrosis, mucositis and trismus (4).

Over the last decade, induction chemotherapy has been advocated, since it may predict tumor’s sensitivity and thus help decide between radical surgery or CR; reduce tumor volume, allowing surgery in tumors otherwise considered inoperable or too mutilating, with the goal of increasing patient’s quality of life; and, more importantly, can erradicate micrometastasis (5). In fact, overall survival in patients with high risk tumors (N2b-N3) treated with induction chemotherapy followed by CR was higher when compared to those treated only with CR (5). Due to its high toxicity profile or risk of compromising the following treatment with CR and controversial results in literature, induction chemotherapy either followed by CR or surgery is considered an option, and without superior effectiveness to conventional treatment of LAOC (2,5,6) Since our patient’s tumor was inoperable, the patient was included in a clinical trial of induction chemotherapy in LAOC, with the perspective of evaluating potential ressectability considering its response. Although tumor volume was frankly diminished after treatment, consolidation with CR was performed by patient’s choice. Vascular supply to the tongue is provided by lingual, facial, pharyngeal and collaterals, making tongue necrosis, especially bilateral, a rare clinical entity (7).

A wide range of etiologies has been described, such as giant cell arteritis (GCA), following the use of vasoconstrictor agents, malignant tumors, neck dissection, shock and infections like syphilis (7). There is a small amount of case reports in literature of tongue necrosis as a complication of radiotherapy (RT).

In light of the clinical presentation of tongue necrosis, a few differential diagnoses were taken into account, such as neoplasms, vasculitis and infections. The major differential diagnosis was with Giant Cell arteritis. Tongue necrosis is a known complication of GCA, a chronic vasculitis of large and medium vessels, but it is usually unilateral (7).

Diagnosis criteria include three out of the five following criteria: age>50 at disease onset, new onset of localized headache, abnormal temporal artery with tenderness or decreased pulse, erythrocyte sedimentation rate>50mm/hr and necrotizing arteritis with predominant mononuclear cell infiltrate or granulomatous process with multinucleated giant cells (7). In our patient, GCA was an unlikely diagnosis, given its bilateral nature and the absence of diagnostic criteria other than age over 50 and high erythrocyte sedimentation rate. Cancer relapse or infectious diseases were also dismissed following laboratory, imaging and histopathologic findings. By exclusion of other diagnosis, we considered it to be a post-RT late complication. There is no consensus on the best treatment, although there is a trend for a conservative approach.(8) Depending on etiology, this may include corticotherapy, antiplatelet and anticoagulant drugs (8).

Surgical treatment includes debridment and healing by secondary intention for smaller necrotic areas or glossectomy with primary closure for larger necrotic areas, to aid with healing and rehabilitation (7).

Full epithelization of the tongue can also be achieved with a conservative approach, with natural auto-amputation and secondary healing (7,8). Since deformity is dependent of the area affected, a higher deformity should be expected with a higher necrotic area (7).Head and neck cancer patients are at a higher risk of developing vascular complications, either as a result of the disease itself or as a result of treatment (9). Radiation damage includes ischemic necrosis and fibrosis of the vasa vasorum, adventitial fibrosis with narrowing and acceleration of the atherosclerotic process, making occlusive vascular disease a well-known late complication of radiotherapy (9). Higher dosages are associated with post-RT vascular disease, but time elapsed after treatment may also play a role (9,10). In fact, the risk for a major cardio and cerebrovascular event increases significantly after 5 years of RT (9). Nonetheless, control of other known cardiovascular risk factors such as hypercholesterolemia, arterial hypertension, diabetes or smoking, must be accomplished to reduce the overall risk of a major event. Medications like statins and angiotensine-converting enzyme also appear to be beneficial in post-RT vascular disease (10). *Boulet et al* (11) found that statin-use reduced the risk of stroke alone by 30% and postulates the hypothesis of lowering the threshold for statin use in these patients. There are currently no guidelines for treatment or prevention of post-RT vascular disease (11). 

It seems beneficial to make a routine non-invasive assessment with carotid bruit auscultation and imaging (with ultrasonography, magnetic resonance angiography, or computed tomographic angiography) (1). Biomarkers of vascular injury, like myeloperoxidase or fibrinogen, may also be indicated (12). 

## Conclusion

Tongue necrosis is a rare complication of post-RT small-vessel disease that may reflect future risk of major-vessel disease such as stroke or even death, highlighting the importance of routine risk assessment while pondering the benefits of prophylaxis with statins or other medications.
